# Accelerometer data collected with a minimum set of wearable sensors from subjects with Parkinson’s disease

**DOI:** 10.1038/s41597-021-00830-0

**Published:** 2021-02-05

**Authors:** Jean-Francois Daneault, Gloria Vergara-Diaz, Federico Parisi, Chen Admati, Christina Alfonso, Matilde Bertoli, Edoardo Bonizzoni, Gabriela Ferreira Carvalho, Gianluca Costante, Eric Eduardo Fabara, Naama Fixler, Fatemah Noushin Golabchi, John Growdon, Stefano Sapienza, Phil Snyder, Shahar Shpigelman, Lewis Sudarsky, Margaret Daeschler, Lauren Bataille, Solveig K. Sieberts, Larsson Omberg, Steven Moore, Paolo Bonato

**Affiliations:** 1 Department of Physical Medicine and Rehabilitation, Harvard Medical School, Spaulding Rehabilitation Hospital, Boston, Massachusetts USA; 2 Department of Rehabilitation and Movement Sciences, Rutgers University, Newark, New Jersey USA; 3 Intel Corporation, IT Advanced Analytics, Bethlehem, Israel; 4 Department of Neurology, Icahn School of Medicine at Mount Sinai, New York, New York USA; 5 Department of Neurology, Harvard Medical School, Massachusetts General Hospital, Boston, Massachusetts USA; 6 Sage Bionetworks, Seattle, Washington 98121 USA; 7 Department of Neurology, Harvard Medical School, Brigham and Women’s Hospital, Boston, Massachusetts USA; 8 Michael J Fox Foundation, New York, New York USA; 9 School of Engineering and Technology, Central Queensland University, Rockhampton, Australia; 10 Wyss Institute for Biologically Inspired Engineering, Harvard University, Cambridge, Massachusetts USA

**Keywords:** Parkinson's disease, Parkinson's disease

## Abstract

Parkinson’s disease (PD) is a neurodegenerative disorder associated with motor and non-motor symptoms. Current treatments primarily focus on managing motor symptom severity such as tremor, bradykinesia, and rigidity. However, as the disease progresses, treatment side-effects can emerge such as on/off periods and dyskinesia. The objective of the Levodopa Response Study was to identify whether wearable sensor data can be used to objectively quantify symptom severity in individuals with PD exhibiting motor fluctuations. Thirty-one subjects with PD were recruited from 2 sites to participate in a 4-day study. Data was collected using 2 wrist-worn accelerometers and a waist-worn smartphone. During Days 1 and 4, a portion of the data was collected in the laboratory while subjects performed a battery of motor tasks as clinicians rated symptom severity. The remaining of the recordings were performed in the home and community settings. To our knowledge, this is the first dataset collected using wearable accelerometers with specific focus on individuals with PD experiencing motor fluctuations that is made available via an open data repository.

## Abstract (summary)

**Table:** 

Measurement(s)	body movement coordination trait • Movement Disorder Society Unified Parkinson’s Disease Rating Scale Questionnaire • Medication • motor coordination/balance trait • sleep pattern • MDS-UPDRS Tasks and Simulated Activities of Daily Living (in-clinic) • Activity of Daily Living
Technology Type(s)	Accelerometer • body movement/behavior method • Clinical Observation • smartphone • Subject Diary
Factor Type(s)	age of patient • gender of patient • timing of medication intake
Sample Characteristic - Organism	Homo sapiens

Machine-accessible metadata file describing the reported data: 10.6084/m9.figshare.13342055

## Background & Summary

Parkinson’s disease (PD) is a progressive neurodegenerative disorder that leads to severe impairments in quality of life due to both motor and non-motor symptoms. The prevalence of PD is estimated to be between 100 and 200/100,000^1–3^; and as the world population ages, the number of cases is expected to increase significantly^4^. While current treatment options include several types of medication and surgical options, the gold standard, L-3,4-dihydroxyphenylalanine (L-dopa), has remained the same over the last 50 years^5^.

L-dopa has been shown to effectively manage the severity of most motor symptoms of PD. Unfortunately, prolonged use of L-dopa can lead to unwanted side-effects termed motor fluctuations that are marked by wearing off, the abrupt loss of efficacy and re-emergence of tremor or bradykinesia prior to the next medication dose, and dyskinesias (involuntary writhing movements). In fact, the incidence of dyskinesia has been estimated at 30–50% five years after initiation of L-dopa treatment and 60–100% 10 years after treatment initiation^6–8^. Motor fluctuations produce substantial disability, lead to impaired quality of life, increased financial burden on the already overstressed healthcare system, and frequently make it challenging to identify an effective pharmacotherapy and titrate patients’ medications accordingly^9–11^. Medication intakes are commonly scheduled in order to minimize off states and dyskinesias^12^. In order to minimize motor fluctuations and their deleterious effects, there is a need for gaining a better understanding of their characteristics. Current methodologies for assessing dyskinesia and fluctuations in motor symptoms mainly rely on clinical scales and patient diaries^13–18^. However, these are limited in their ability to capture longitudinal and real-world measures. The ubiquitous nature of wearable technology opens up the possibility for longitudinal monitoring in the home and community setting.

**Fig. 1:**
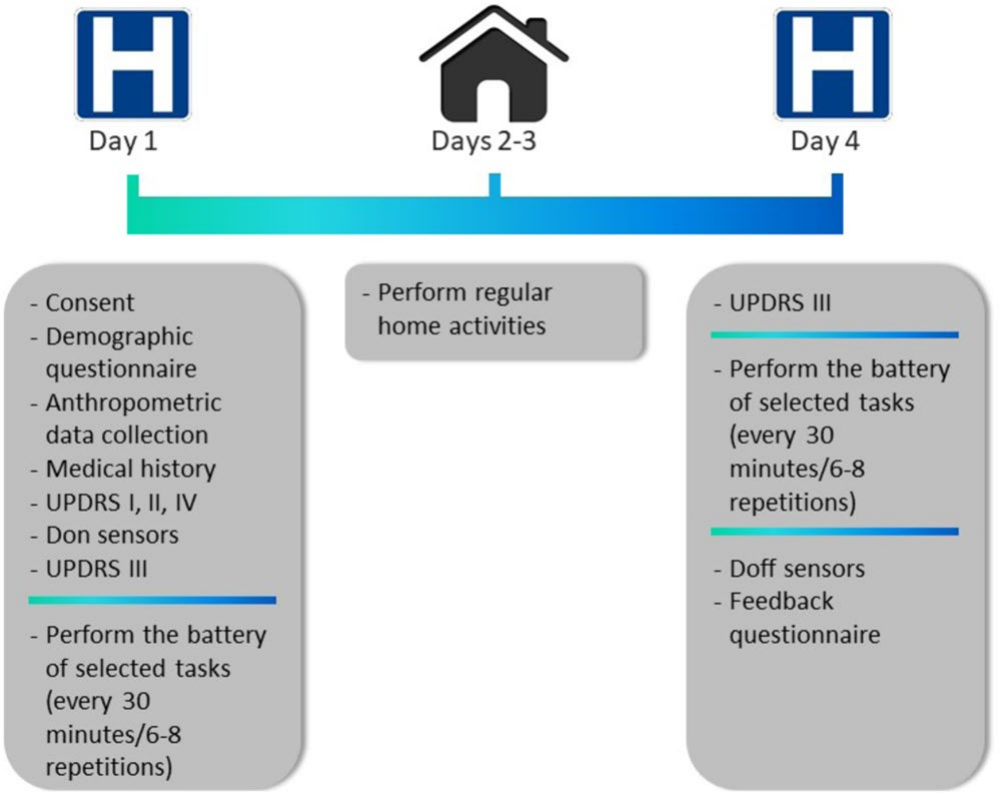
Illustration of the study protocol. Data collection started in the laboratory on Day 1 where a battery of motor tasks was performed multiple times by subjects and questionnaires were filled out. Data collection continued on Days 2 and 3 in the home setting and was completed in the laboratory on Day 4.

**Fig. 2:**
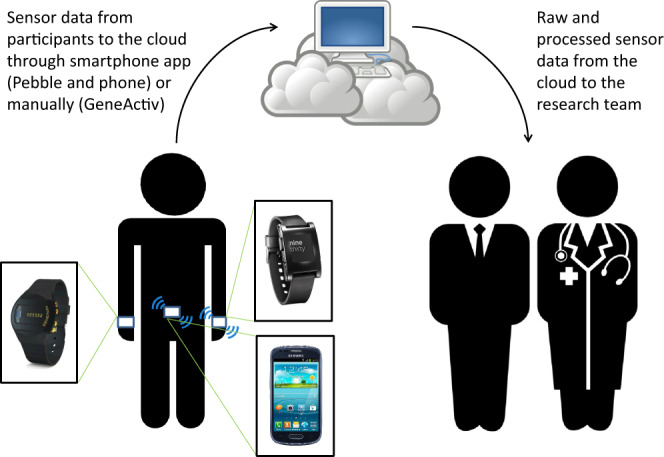
Illustration of the sensor placement and cloud platform. Study volunteers donned a GeneActiv device on their most affected wrist, a Pebble smartwatch on their least affected wrist and a smartphone at the waist. Real-time data from the sensors embedded in the Pebble smartwatch and the smartphone were transmitted to a cloud platform through a custom-designed smartphone application. The sensor data could be visualized in real time by research staff. After completion of the protocol, the GeneActiv data was manually uploaded to the cloud platform. The combined data could then be accessed by researchers to perform analyses to be shared with the research team.

In order to derive measures that can be used for research, patient care, and clinical trials, we need algorithms to estimate the severity of motor symptoms and dyskinesia from data collected using wearable technology. We posited that a suitable way to achieve this goal was to collect data in the laboratory in order to have ground truth labels to assess the accuracy of algorithms and then to collect data in the home setting to assess feasibility of using such measures outside of the laboratory and compare the outcomes of both data collection paradigms. To this end, we developed the Levodopa Response Study. The study design is shown in Fig. 1. Study participants were recruited from 2 sites, Spaulding Rehabilitation Hospital (Boston, MA) and Mount Sinai Hospital (New York, NY). Subjects recruited in the study came to the laboratory on Day 1, while on their usual medication schedule, where they donned multiple sensors (GeneActiv device on the most affected upper limb, Pebble smartwatch on the least affected upper limb, and a Samsung Galaxy S3 Mini smartphone in a fanny pack worn in front at the waist) (Fig. 2). This sensor arrangement was devised as the minimum number of sensors we hypothesized would be necessary to capture whole-body information regarding the presence and severity of motor fluctuations. These specific devices were chosen for the purpose of testing different consumer-grade technologies and form-factors which could be easily deployed for continuous monitoring in the field.

We know from previous studies that limb-specific symptom severity requires a larger number of sensors^19–22^, but also that using multiple sensors increases patient burden and may not be feasible for long-term monitoring of patients participating in clinical trials or for disease management. For this reason, many research studies have used a smartphone^23–25^ or a combination of a smartphone and a smartwatch^26^ to collect accelerometer data in the home and community settings and estimate motor symptom severity in individuals with PD. Using a minimum set of sensors does not allow one to monitor motor patterns displayed by individual body segments. However, it is unclear if this is necessary from a clinical point of view. To enable a comparison between monitoring individuals using a minimum set of sensors and monitoring them using sensors on each major body segment (i.e. upper limbs, lower limbs, and trunk), subjects recruited at Spaulding Rehabilitation Hospital were also outfitted with a set of five Shimmer 3 sensors (Shimmer Research Ltd, Dublin, Ireland), one on each limb and one on the pelvis. This subset of data is described in detail in the companion paper^27^.

During the laboratory visit, subjects first performed section III of the MDS-UPDRS^28^. Thereafter, to assess symptom fluctuations over a motor fluctuation cycle, subjects performed a battery of motor tasks that included some activities of daily living (ADL) and selected items of section III of the MDS-UPDRS. This battery of tasks lasted approximately 20 minutes and was repeated 6–8 times at 30-minute intervals. The protocol included rest periods. Subjects were encouraged to take breaks as needed throughout the study procedures. Afterward, subjects were sent home and instructed to wear the sensors for two days while conducting their usual activities. On Day 4, subjects returned to the laboratory and performed the same set of tasks as they did on Day 1. This time they were asked to arrive at the laboratory in a practically-defined off medication state (i.e., medications were withheld overnight for approximately 12 hours), and to take their medications after the first repetition of the above-mentioned battery of tasks, in order to assess the full range of symptoms and medication side-effects. At completion of the data collection, subjects doffed the sensors.

This dataset comprises wearable sensor data collected in the laboratory with ground truth labels of symptom severity during the performance of scripted motor tasks as well as wearable sensor data collected in the home setting during the performance of unscripted motor tasks. To our knowledge, this is the first dataset collected using wearable sensors in individuals with PD experiencing motor fluctuations that is made available to the scientific community via an open data repository. The dataset complements other publicly available wearable sensor datasets such as the mPower study^29^ and the Daphnet Freezing of Gait Dataset^30^. These datasets also consist of wearable sensor data collected from subjects with PD during the performance of motor tasks. However, the dataset herein presented has the added value of including clinician-assessed ground truth labels of symptom severity, focusing solely on individuals with PD experiencing motor fluctuations, and including a wide variety of motor tasks and additional sensors, as well as continuous recording over 4 days (see the Methods section for some exceptions). The continuous at-home monitoring data can inform researchers and clinicians on the variability of symptom severity and the characteristics of motor fluctuations that subjects experience daily. The dataset can be used not only to develop robust algorithms for the estimation of symptom severity, but also for activity recognition in individuals with PD and potentially for other clinically relevant applications. A portion of the dataset herein presented was recently utilized for the Parkinson’s Disease Digital Biomarker DREAM Challenge that aimed to identify the best data analysis methods to extract features from wearable sensor data for the estimation of the presence of different symptoms as well as their severity^31^. The data challenge showed that this dataset has great potential for enabling important advances in the management of symptoms and motor fluctuations in PD.

## Methods

### Participants

**Fig. 3:**
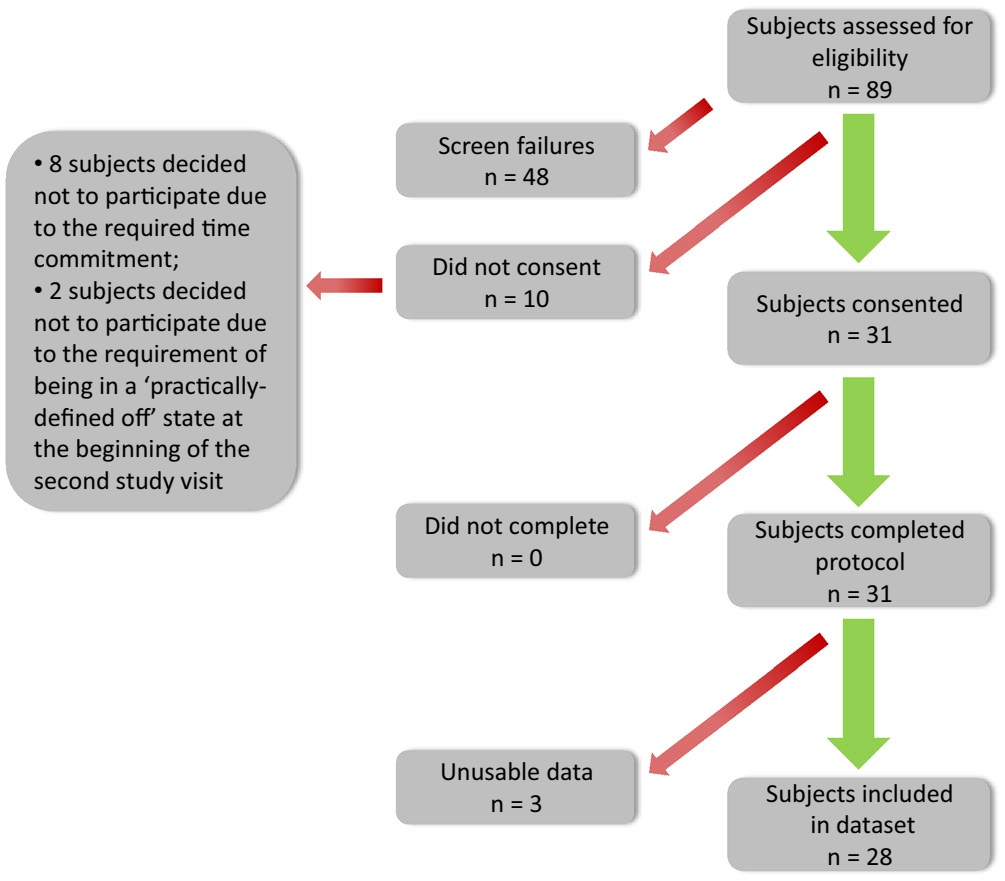
Illustration of subject onboarding. Note that 48 screened subjects did not meet the study inclusion criteria, 10 subjects who met the study inclusion criteria did not consent due to time commitment or having to come to the laboratory in a practically-defined off state, and all subjects that provided consent completed the protocol. However, data from 3 subjects were excluded from the dataset because of protocol change (n = 2) and a considerable amount of missing data (n = 1).

A total of 31 subjects with PD, who self-reported to experience motor fluctuations, with Hoehn & Yahr scores ranging from II to IV (ordinal scale of symptom progression)^28^, were enrolled in the study (see Fig. 3). Subjects were recruited and enrolled at two study sites: Spaulding Rehabilitation Hospital (n = 19) and Mount Sinai Hospital (n = 12). All subjects signed the informed consent form. Inclusion criteria were: (1) community dwelling men and women between 30 and 80 years of age with diagnosed PD, (2) currently taking L-dopa, (3) reporting at least mild dyskinesia, (4) reporting motor fluctuations, and (5) being able to operate a smartphone. Exclusion criteria were: (1) having other serious neurological condition (e.g. clinically significant stroke, brain tumor, hydrocephalus, epilepsy, other neurodegenerative disorder, encephalitis, repeated head trauma), or (2) having deep brain stimulation (DBS). All procedures were approved by the Institutional Review Board of both study sites (Spaulding IRB # 2014P000847; NY IRB # 14-1569). While 31 subjects were enrolled and completed the study protocol (see below), data for two subjects from Spaulding Rehabilitation Hospital and one from Mount Sinai Hospital were excluded (Fig. 3). Data from the first two individuals enrolled in the study was excluded because some of the motor tasks initially adopted in the study were modified according to issues encountered during the first assessments. Data for a third participant was excluded due to substantial quantity of missing data which was the result of a technical malfunction of the sensors.

### Data collection

**Fig. 4:**
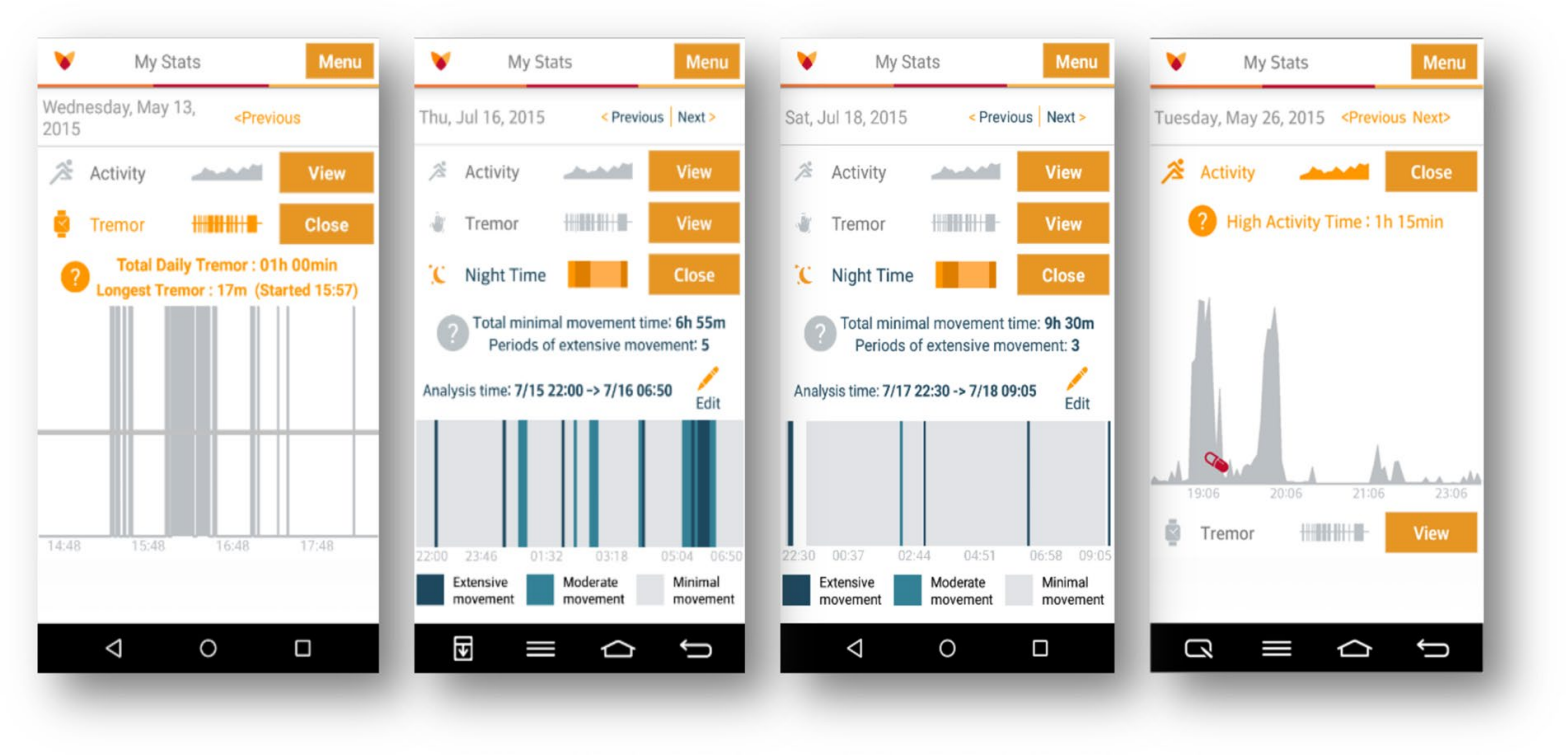
Smartphone application graphical user interface. Subjects were able to input information using the interface and were able to visualize the data from the Pebble watch and the phone.

Study volunteers were asked to wear a GeneActiv wristwatch on their most affected side, a Pebble smartwatch on their least affected side, and a smartphone at the waist. The GeneActiv wristwatch provides continuous accelerometer data and was set to collect data in the range ±8 G, at a sampling rate of 50 Hz. Custom-designed applications enabled the recording of continuous accelerometer data also from the Pebble-embedded sensor in the range ±4 G, also at a sampling rate of 50 Hz and from the smartphone-embedded sensor in the range ±2 G, at a sampling rate of 50 Hz. Specifically, the Michael J. Fox Foundation for Parkinson’s Research partnered with Intel® Corporation to develop the Fox Wearable Companion App (FWC App), a mobile and wearable application for PD research (see Fig. 4 for examples of the smartphone application user interface components). The FWC App is part of the Intel® Pharma Analytics Platform, an edge to cloud artificial intelligence solution that enables remote monitoring and continuously captures clinical data from study subjects using a variety of sensors, including wearable devices. In the study, accelerometer data collected using the smartwatch was streamed to the FWC App via Bluetooth and transmitted, together with the accelerometer data recorded from the smartphone-embedded sensor, to the cloud platform using an internet connection (Wi-Fi or mobile network). The FWC App provided also the ability to record patients’ reported outcomes, such as symptom severity, on/off state, and medication intake. It also provided the ability to perform and display a limited set of data aggregations and analytics, such as activity level during waking and sleeping hours. However, these features of the FWC App were not utilized in the current study. The cloud platform consisted of a scalable system where data could be securely stored and managed as well as processed using APIs for advanced data analytics.

Even though there is evidence in the literature that additional sensors (e.g., gyroscopes) could provide useful information for tracking symptoms and motor complications^32–35^, we collected only accelerometer data to maximize the duration of the recordings without having to recharge the units and hence minimize subjects’ burden caused by the need to recharge the devices.

A summary of all the available data is shown in Table 1 and further details are provided in the following tables: Table 2 - Metadata of Patient Onboarding^36^, Table 3 - Metadata of Laboratory Visits^36^, Table 4 - Task Scores – Part I^36^, Table 5 - Sensor Data – Part I^36^, Table 6 - Subject Diary^36^, Table 7 - Sleep Diary^36^, Table 8 - Medication Diary^36^, and Table 9 - Feedback Survey^36^. Table 10 and Online-only Table 1 shows the duration and the percentage of valid data for each sensing device and for each subject for the entire data collection as well as the percentage of valid data for the laboratory visits and the recordings in the home and community settings.

**Table 1:** Summary available data.

Task name	Type of task and schedule	Table ^Reference^
Metadata of Patient Onboarding	Survey – Once	Table 2^36^
Metadata of Laboratory Visits	Survey – Twice	Table 3^36^
Task Scores – Part I	Assessment - Twice	Table 4^36^
Sensor Data – Part I	Activity - Four days	Table 5^36^
Subject Diary	Survey - Twice	Table 6^36^
Sleep Diary	Survey - Once	Table 7^36^
Medication Diary	Survey - Once	Table 8^36^
Feedback Survey	Survey - Once	Table 9^36^
Duration and Percentage of Valid Data (Total)	Not Applicable	Table 10
Duration and Percentage of Valid Data (Laboratory visits and at-home period)	Not Applicable	Online-only Table 1

**Table 2:** Metadata of Patient Onboarding.

Variable name	Variable details	Variable unit
subject_id	Alphanumeric	
cohort	PD	
gender	One of {‘Male’, ‘Female’}	
birth_year	Integer	year
dominant_hand	One of {‘Right’, ‘Left’}	
upper_limb_length	Real number	cm
upper_arm_length	Real number	cm
lower_arm_length	Real number	cm
lower_limb_length	Real number	cm
thigh_length	Real number	cm
shank_length	Real number	cm
height	Real number	cm
weight	Real number	kg
visit_date	Date	
diagnosis_day	Integer	day
diagnosis_month	Integer	month
diagnosis_year	Integer	year
pd_most_affected_side	One of {‘Right’, ‘Left’, ‘Bilateral’}	
gait_impediments	Boolean	
posture_instability	Boolean	
tremor	Boolean	
bradykinesia	Boolean	
disrupted_sleep	Boolean	
freeze_of_gait	Boolean	
dyskinesia	Boolean	
rigidity	Boolean	
other_symptoms	Text	
last_levodopa_dose_timestamp	Integer	
regular_medication	Text	
geneActive_num	Integer	
pebble_num	Alphanumeric	
geneActive_hand	One of {‘Right’, ‘Left’}	
pebble_hand	One of {‘Right’, ‘Left’}	
smartphone_location	One of {‘Right’, ‘Left’}	
recording_start	Time	
recording_end	Time	
timezone	Text	
updrs_time	Time	
updrs_score_p1	Integer	
updrs_score_p2	Integer	
updrs_score_p3	Integer	
updrs_score_p4	Integer	
h_and_y_score	Integer	
updrs_second_visit_time	Time	
updrs_second_visit_score_p3	Integer

**Table 3:** Metadata of Laboratory Visits.

Variable name	Variable details
subject_id	Alphanumeric
visit	One of {‘1’, ‘2’}
clinical_assessment_timestamp	Integer
medication_intake_timestamp	Integer
medication_name	Text
medication_dosage	Text
timezone	Text
second_medication_intake_timestamp	Integer
stopwatch_start_timestamp	Integer
fox_insight_app_start_timestamp	Integer
geneActiv_start_timestamp	Integer
general_comments	Text

**Table 4:** Task Scores – Part I.

Variable name	Variable details
subject_id	Alphanumeric
visit	Integer
session	Integer
task_id	Integer
task_code	One of {‘stndg’, ‘wlkgs’, ‘wlkgc’, ‘strsu’, ‘strsd’, ‘wlkgp’, ‘drawg’, ‘ftnr’, ‘ftnl’, ‘ramr’, ‘raml’, ‘ststd’, ‘typng’, ‘ntblt’, ‘drnkg’, ‘orgpa’, ‘fldng’, ‘sittg’}
repetition	One of {‘1’, ‘2’}
timestamp_start	Real number
timestamp_end	Real number
phenotype	One of {‘tremor’, ‘dyskinesia’, ‘bradykinesia’}
body_region	One of {‘RightUpperLimb’, ‘LeftUpperLimb’, ‘LowerLimbs’}
score	One of {‘Yes’, ‘No’, ‘NotApplicable’, ‘0’, ‘1’, ‘2’, ‘3’, ‘4’}

**Table 5:** Sensor Data – Part I.

Variable name	Variable details
subject_id	Alphanumeric
device	One of {‘GENEActiv’, ‘Pebble’, ‘Phone’}
device_position	One of {‘LeftUpperLimb’, ‘LowerLimbs’, ‘RightUpperLimb’}
participant_day	Integer
timestamp_start	Real number
timestamp_end	Real number
source_file	Hyperlink
data_file_handle_id	Hyperlink

**Table 6:** Subject Diary.

Variable name	Variable details
subject_id	Alphanumeric
participant_day	One of {‘2’, ‘3’}
session_label	Text
session_number	Integer
off	Boolean
dyskinesia	Boolean
troublesome_dyskinesia	Boolean
tremor	Boolean
freeze_of_gait	Boolean
slowness_of_movements	Boolean
comments	Text

**Table 7:** Sleep Diary.

Variable name	Variable details
subject_id	Alphanumeric
entry_id	Integer
sleep_event_date	Date
sleep_event_hour	Time
timestamp	Integer
sleep_event_type	One of {‘fall_asleep_time’, ‘wake_up_time’}

**Table 8:** Medication Diary.

Variable name	Variable details
subject_id	Alphanumeric
med_id	Integer
med_timestamp_date	Date
med_timestamp_hour	Time
timestamp	Integer
pd_related_meds_taken	Text
other_meds_taken	Text

**Table 9:** Feedback Survey.

Variable name	Variable details
subject_id	Alphanumeric
charge_smartphone	Integer
charge_pebble	Integer
experience_watches	Integer
experience_devices	Integer
clearness_diary	Integer
accuracy_diary	Integer
additional_feedback_device_phone	Text
additional_feedback_diary	Text
additional_feedback_experiment	Text

**Table 10:** Total Duration and Percentage of Valid Data.

Subject ID	Duration (hours)	% Valid Data		
		GeneActiv	Pebble	Phone
3_BOS	76.96	100.00%	61.60%	77.70%
4_BOS	77.14	100.00%	0.00%	0.00%
5_BOS	78.80	100.00%	77.00%	15.80%
6_BOS	78.94	100.00%	58.30%	10.80%
7_BOS	78.45	100.00%	95.40%	94.90%
8_BOS	78.09	100.00%	96.70%	96.40%
9_BOS	77.00	100.00%	98.20%	97.80%
10_BOS	76.23	100.00%	66.70%	89.10%
11_BOS	76.71	100.00%	60.70%	96.80%
12_BOS	77.94	100.00%	70.30%	71.10%
13_BOS	76.69	100.00%	67.10%	99.30%
14_BOS	77.13	100.00%	76.40%	80.90%
15_BOS	76.45	100.00%	72.60%	89.40%
16_BOS	76.88	100.00%	99.80%	99.10%
17_BOS	78.70	100.00%	41.50%	3.40%
18_BOS	76.46	100.00%	79.90%	18.40%
19_BOS	76.71	100.00%	38.10%	38.80%
2_NYC	74.21	100.00%	39.80%	7.40%
3_NYC	73.78	100.00%	94.20%	20.00%
4_NYC	72.67	100.00%	55.20%	17.80%
5_NYC	73.54	100.00%	74.80%	20.80%
6_NYC	74.18	100.00%	100.00%	99.40%
7_NYC	74.92	100.00%	100.00%	99.40%
8_NYC	75.97	100.00%	99.30%	98.80%
9_NYC	75.43	100.00%	93.50%	93.00%
10_NYC	72.90	88.10%	18.60%	80.20%
11_NYC	72.53	100.00%	19.40%	70.50%
12_NYC	74.84	100.00%	79.90%	98.00%

Accelerometer data was generally collected over 4 consecutive days^36^. However, for four of the subjects recruited in the New York site (3_NY; 4_NY; 10_NY; 11_NY) there was a gap between the data collected during Day 3 (in-home) and the data collected during Day 4 of the study (second laboratory visit) because of scheduling issues. In other words, for some subjects, it was not possible to schedule the second lab visit 3 days after initiating the study and hence data was collected for additional days before study volunteers were able to come to the laboratory. Data collected between the end of Day 3 and the beginning of the day of the second laboratory visit (herein referred to as Day 4) was discarded so that all the subjects had 4 days of data recordings. For subject 10_NY, the gap between Day 3 and the day of the second laboratory visit was approximately 19 days. The GeneActiv sensor, which could not be charged at home, stopped recording data during the gap period and had to be replaced with a new unit in the laboratory just before the beginning of the second laboratory visit. In contrast, the Pebble smartwatch and the smartphone kept recording for the entire period as the subject recharged them periodically. Furthermore, for subject 11_NY, the research team swapped the GeneActiv from the left wrist to the right wrist between the first and the second session (after 36 min and 34 s of data recording).

Acceleration in the three cartesian coordinate axes, as well as the vector magnitude are reported for the three devices in m/s^2^. Sensor data timestamps are reported using a Unix epoch timestamp notation, from which it is possible to derive the exact date and time of each data sample. In addition to the acceleration data, clinical labels of symptom severity and symptom presence are provided for each limb and each motor task^36^ as generated by a clinician during the in-laboratory sessions^36^. Limb-specific (i.e. left upper limb, right upper limb, and lower limbs) tremor severity scores (0–4) are provided, as well as upper-limb and lower-limb presence of dyskinesia (yes or no) and bradykinesia (yes, no, or not applicable).

### Protocol

#### Day 1: In-clinic monitoring

On the first day of data collection, subjects came to the laboratory in an on medication state, answered demographic as well as medical history questions, were measured and weighed, and donned the sensors on specific limbs^36^. Clinical symptoms were assessed using the Movement Disorders Society Unified Parkinson’s Disease Rating Scale (MDS-UPDRS)^36^. Then, subjects performed the first testing block which comprised several motor tasks. These motor tasks are standing (stndg), walking in a straight line for 30 s (wlkgs), walking in a straight line for 30 s while counting backwards (wlkgc), walking up the stairs (strsu), walking down the stairs (strsd), walking through a narrow corridor (wlkgp), finger-to-nose for 15 s (twice with each arm) (ftnr and ftnl), alternating hand movements for 15 s (twice with each arm) (ramr and raml), drawing (drawg), typing on a keyboard for 30 s (typng), opening a bottle and pouring water (three times) (drnkg), arranging sheets of paper in a folder (twice) (orgpa), sit-to-stand-to-sit (three times) (ststd), assembling nuts and bolts for 30 s (ntblt), folding a towel three times (fldng), and sitting (sittg)^36^. This testing block (except for walking up and down the stairs) was repeated every 30 minutes for 3 to 4 hours (6 to 8 repetitions). During each task, a trained clinician assessed the severity (tremor) or presence (bradykinesia and dyskinesia) of symptoms for each limb separately^36^. Note that the clinical researchers who provided the clinical scores were all MDS-UPDRS certified. Also, we organized the rating task so that a given clinical researcher provided symptom scores for all the repetitions of a given task.

At the end of the visit, subjects were instructed on how to use the custom smartphone application and on how and when to remove, recharge, and put the devices back on. They were provided with a printed at-home instruction manual, containing detailed descriptions of the procedures that were explained to them in the laboratory.

#### Days 2-3: At-home monitoring

Once the laboratory data collection was completed, subjects went home while still wearing the sensors. Subjects wore the sensors at home for 2 complete days while they performed their usual daily activities. The GeneActiv sensor did not need to be recharged and could be worn at all times. The Pebble sensor and the smartphone required to be charged once a day (participants were instructed to charge these two devices preferably at night). The GeneActiv and the Pebble sensors were water-resistant, and it was possible for study participants to keep them on while showering. Subjects also recorded medication intake times and doses, and the sleep and wake up times^36^.

#### Day 4: In-clinic monitoring

On Day 4, study volunteers returned to the laboratory, but this time in an off medication state (i.e. without taking their regularly scheduled morning dose of medication/s which led to a minimum medication withdrawal time of 10 hours with the majority of subjects having withheld medication/s for at least 12 hours). A portion of the procedures carried out during the data collection on Day 1 were repeated, starting with section III (motor assessment) of the UPDRS^36^. After the first testing block, that was carried out off medications, subjects took their regularly scheduled morning medication dose and the following 5 to 7 repetitions of the above-mentioned battery of tasks were completed according to the same timeline as on Day 1 (i.e. every 30 minutes)^36^. The same trained clinical researchers provided scores for symptom severity or presence/absence clinical scores for all the repetitions of all the tasks^36^. Note that the same clinical researcher provided symptom severity for both the in-clinic sessions of a given subject (i.e. for both the Day 1 and Day 4 sessions). However, different clinical researchers examined different subjects. Once completed, subjects doffed the sensors and were asked to complete a feedback questionnaire regarding the use of the technology^36^.

It is worth noting that two participants deviated from the protocol: one subject (4_BOS) arrived in the off state to the laboratory on Day 1 and thus was asked to arrive in the on state on Day 4. Another subject (3_BOS) had a medication intake before the beginning of the second laboratory visit (Day 4) (4 hours before the session) and therefore was not in a practically-defined off state.

Using this protocol, we recorded approximately 4 days of continuous wearable sensor data (Table 5^36^); including 12 to 16 laboratory testing blocks containing data recorded during the performance of specific motor tasks^36^ across a range of medication states in individuals with PD exhibiting motor fluctuations. The specified motor tasks were labeled with clinical symptom severity or presence/absence scores (Table 4^36^). In addition, we captured 2 days of unlabeled home data. We also captured demographic and clinical data (Table 2^36^), as well as patient self-reported diaries (Tables 6, 7 and 8^36^) and feedback regarding the use of the technology (Table 9^36^).

### Data pre-processing

The raw sensor data from the GeneActiv, Pebble and smartphone was pre-processed in order to achieve the following objectives:

1. Identify intervals with missing data in the raw signals
2. Resample the time series with a sampling rate of 50 Hz
3. Temporally align the signals collected using different devices

All the pre-processing and alignment procedures were carried out using MATLAB (Mathworks, Natick, MA).

First, the raw data collected using each device was processed by filling gaps in the time series due to missing data. Gaps were filled using sequences of NaN’s (Not-a-Number’s, i.e., not valid values), according to the original sampling rate (i.e., 50 Hz). This allowed us to represent the signals as time series for the entire duration of the data collection, with timestamp vectors containing only increasing valid values and acceleration signals including both valid and invalid data corresponding to valid and missing values in the original raw signals, respectively. At this point, each time series could still be marked by uneven sampling intervals. Hence, we carried out a resampling procedure using a linear interpolation method thus resulting in an evenly sampled time series with a sampling rate exactly equal to 50 Hz. Then, the resampled signals were temporally aligned by exploiting a simultaneous physical “shake” of all the devices that was done at the beginning of the first session (Day 1) and at the end of the last session (Day 4) in the clinic. The “shake” event consisted in intense upward/downward movements of all devices held together. This was done by a member of the research staff in the laboratory. The event was associated with an easily distinguishable pattern in the accelerometer time series of each device, which enabled the extraction of temporal offsets between the reference sensor (the GeneActiv device) and the other two devices at the two different points in time when the “shake” event occurred (beginning of the laboratory session on Day 1 and end of the laboratory session on Day 4). This was achieved by using a cross-correlation based algorithm. Since the internal clocks of the devices were subject to drift, the offsets on Day 1 could be slightly different from those on Day 4. In order to address this issue, the magnitude of the drift was computed from the difference between the offsets on Day 1 and Day 4 for the non-reference devices. Then, the drift was removed under the assumption that it developed linearly during the entire data collection. The drift-free time series of the Pebble smartwatch and the smartphone were obtained by removing this linear trend from their time axis. Finally, the time alignment between all devices was achieved by shifting the timestamp vector of the non-reference devices by the offsets computed for Day 1. The correctness of this procedure was visually validated by checking the alignment of the sensor data corresponding to the “shake” event on Day 4. The results showed an error in the time synchronization among devices that was always below 100 ms, which was considered negligible. The resulting aligned signals were then saved and posted on Synapse.org.

### Dataset descriptive statistics

**Fig. 5:**
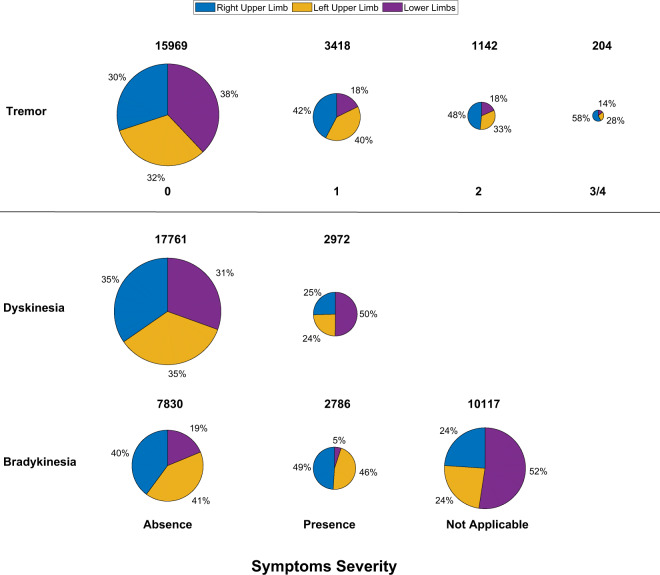
Distribution of the clinical scores for (**a**) tremor, (**b**) dyskinesia, and (**c**) bradykinesia related to the motor tasks performed during the laboratory visits.

A total of 6911 tasks were performed during the two laboratory visits by the 28 subjects whose data is shared as part of this manuscript. The distributions of the clinical scores assigned to all the motor tasks performed during the laboratory visits are shown in Fig. 5. The number of instances for both the upper limbs and the lower limbs (combined) contributing to the total amount of clinical scores for each severity or presence/absence of symptoms is highlighted (with different colors) in both the pie charts and in the text labels.

Table 10 shows the total duration of the collected data and the overall percentage of valid data for each sensing device and for each subject. Detailed information on the duration and the percentage of valid data for the two laboratory visits and the recordings in the home and community settings are provided in Online-only Table 1.

## Data Records

De-identified study data, consisting of questionnaire responses and sensor data (Pebble smartwatch, GeneActiv unit, and smartphone), were exported to Synapse. Synapse is a general-purpose data and analysis sharing service where members can work collaboratively, analyze data, share insights and have attributions and provenance of those insights to share with others. Synapse is developed and operated by Sage Bionetworks.

A total of 31 individuals consented to participate in the study and completed the data collections. Of those participants, 28 had data that could be utilized for analysis and shared. As mentioned above, data from two subjects was excluded due to a change in study protocol, and data from an additional subject was excluded due to excessive quantity of missing data (due to a technical problem with the sensors). All data contributed by study participants are listed in Table 1.

All coded datasets (Table 1^36^) are stored and are accessible via the Synapse platform with associated metadata and documentation and can be accessed at the following URL: 10.7303/syn20681023

## Technical Validation

The data herein provided was collected using devices with proprietary technical validation. Hence, we do not provide test-retest nor other technical validation datasets. However, others have reported technical validation data for the sensors utilized in the study^37,38^. Also, all metadata and sensor data were visually inspected by trained research staff.

## Usage Notes

Researchers who are interested in accessing the data need to complete the following steps:

1. Have a Synapse account (https://synapse.org)
2. Have their Synapse User Profile validated by the Synapse Access and Compliance Team (ACT)
3. Become a Synapse Certified user
4. Submit an Intended Data Use statement
5. Agree to the Conditions for Use associated with each data source (see DOIs for each data source)

While certain data types may have additional Conditions for Use (e.g. clinical scale copyrights), the overarching Conditions for Use are as follows:

- You confirm that you will not attempt to re-identify research participants for any reason, including for re-identification theory research.
- You reaffirm your commitment to the Synapse Awareness and Ethics Pledge.
- You agree to abide by the guiding principles for responsible research use and data handling as described in the Synapse Governance documents.
- You commit to keeping the data confidential and secure.
- You agree to use the data exclusively as described in your submitted Intended Data Use statement.
- You understand that the data may not be used for commercial advertisement or to re-contact research participants.
- You agree to report any misuse or data release, intentional or inadvertent to the ACT within 5 business days by emailing act@sagebase.org.
- You agree to publish findings in open access publications.
- You promise to acknowledge the L-dopa study investigators in all publications and presentations resulting from using the data as follows: “These data were part of the L-dopa study funded by the Michael J Fox Foundation”.

### Download the data

The data are stored in the Synapse data repository and can be accessed with different modalities:

1. Web-based download: the user can individually download each file directly from the web browser;
2. Python, R, and command line clients;
3. REST API.

Additional information and code examples about the data access procedures for this specific dataset can be found at https://www.synapse.org/#!Synapse:syn20681023/wiki.

Generic documentation about the APIs for interacting with Synapse data repositories are available at https://docs.synapse.org/articles/api_documentation.html.

## Data Availability

The only data processing procedures that we performed on the dataset were the ones described above. The first procedure was carried out to temporally align the data collected using different sensors. The second procedure was carried out to obtain an evenly-sampled timeseries.

## Online-only Table

**Online-only Table 1:** Duration and percentage of valid data for the two laboratory visits and the at-home period.

Subject ID	Visit 1 Laboratory (Day 1)				Visit 2 Laboratory (Day 4)				Home			
	Duration (hours)	% Valid Data			Duration (hours)	% Valid Data			Duration (hours)	% Valid Data		
		GeneActiv	Pebble	Phone		GeneActiv	Pebble	Phone		GeneActiv	Pebble	Phone
3_BOS	4.59	100.00%	91.93%	95.71%	4.63	100.00%	96.50%	99.86%	67.74	100.00%	57.12%	75.02%
4_BOS	5.67	100.00%	0.00%	0.00%	3.34	100.00%	0.00%	0.00%	68.14	100.00%	0.00%	0.00%
5_BOS	4.67	100.00%	99.89%	28.31%	5.76	100.00%	59.26%	12.35%	68.37	100.00%	76.94%	15.22%
6_BOS	4.03	100.00%	2.10%	0.00%	6.79	100.00%	41.67%	3.56%	68.12	100.00%	63.28%	12.20%
7_BOS	4.16	100.00%	90.50%	89.99%	6.33	100.00%	49.00%	48.67%	67.96	100.00%	99.99%	99.52%
8_BOS	4.22	100.00%	100.00%	99.44%	5.31	100.00%	54.01%	53.93%	68.55	100.00%	99.82%	99.52%
9_BOS	3.94	100.00%	99.99%	99.47%	4.58	100.00%	70.51%	69.97%	68.48	100.00%	99.98%	99.54%
10_BOS	5.45	100.00%	100.00%	99.43%	4.92	100.00%	13.51%	65.79%	65.86	100.00%	67.93%	90.01%
11_BOS	4.61	100.00%	86.86%	86.42%	4.22	100.00%	0.00%	92.63%	67.88	100.00%	62.69%	97.81%
12_BOS	5.09	100.00%	96.06%	94.89%	4.72	100.00%	86.76%	86.77%	68.13	100.00%	67.23%	68.21%
13_BOS	5.00	100.00%	100.00%	99.31%	3.55	100.00%	100.00%	99.55%	68.14	100.00%	63.02%	99.34%
14_BOS	5.37	100.00%	100.00%	99.49%	3.00	100.00%	99.36%	98.69%	68.76	100.00%	73.56%	78.72%
15_BOS	4.74	100.00%	89.91%	92.22%	4.68	100.00%	89.17%	88.51%	67.04	100.00%	70.16%	89.23%
16_BOS	4.44	100.00%	100.00%	99.46%	3.62	100.00%	100.00%	99.47%	68.82	100.00%	99.73%	99.02%
17_BOS	4.24	100.00%	98.98%	0.00%	5.78	100.00%	8.43%	42.06%	68.68	100.00%	40.77%	0.35%
18_BOS	4.55	100.00%	95.97%	93.70%	4.15	100.00%	64.90%	0.00%	67.76	100.00%	79.74%	14.44%
19_BOS	5.18	100.00%	87.52%	84.84%	4.26	100.00%	67.55%	66.20%	67.28	100.00%	32.41%	33.55%
2_NYC	4.43	100.00%	99.37%	23.03%	4.67	100.00%	98.37%	13.44%	65.10	100.00%	31.55%	5.87%
3_NYC	4.18	100.00%	83.73%	16.00%	3.84	100.00%	96.90%	9.59%	65.77	100.00%	94.73%	20.81%
4_NYC	4.63	100.00%	75.33%	21.83%	3.75	100.00%	98.60%	17.17%	64.29	100.00%	51.19%	17.56%
5_NYC	4.29	100.00%	79.32%	15.68%	3.90	100.00%	100.00%	0.00%	65.35	100.00%	72.97%	22.37%
6_NYC	4.13	100.00%	100.00%	99.36%	3.61	100.00%	100.00%	99.35%	66.44	100.00%	99.98%	99.44%
7_NYC	4.00	100.00%	100.00%	99.36%	2.83	100.00%	100.00%	99.42%	68.09	100.00%	99.99%	99.35%
8_NYC	4.14	100.00%	100.00%	99.45%	4.13	100.00%	88.49%	88.14%	67.69	100.00%	99.97%	99.45%
9_NYC	3.93	100.00%	100.00%	99.35%	5.27	100.00%	74.69%	74.41%	66.24	100.00%	94.65%	94.07%
10_NYC	3.88	100.00%	100.00%	99.19%	2.75	100.00%	100.00%	99.44%	66.27	86.91%	10.51%	78.26%
11_NYC	4.09	100.00%	100.00%	99.43%	3.48	100.00%	92.52%	92.27%	64.97	100.00%	10.42%	67.49%
12_NYC	4.37	100.00%	100.00%	99.31%	3.73	100.00%	100.00%	99.43%	66.74	100.00%	77.43%	97.79%
